# First-principles study of oxygen vacancies in LiNbO_3_-type ferroelectrics†

**DOI:** 10.1039/d4ra00833b

**Published:** 2024-03-18

**Authors:** Jing Li, Xiaohui Liu

**Affiliations:** a School of Physics, Shandong University Ji'nan 250100 China liuxiaohui@sdu.edu.cn

## Abstract

LiNbO_3_-type ferroelectric oxides, as an important class of non-centrosymmetric compounds, have received great attention due to their important and rich properties. Although oxygen vacancies are widely present, studies of them in LiNbO_3_-type ferroelectric oxides are rare. In this article, we consider three representative LiNbO_3_-type ferroelectric oxide materials LiNbO_3_, ZnTiO_3_ and ZnSnO_3_ to study the impact of oxygen vacancy doping using first principles calculations. LiNbO_3_ and ZnTiO_3_ have ferroelectrically active cations Nb^5+^ and Ti^4+^, while ZnSnO_3_ does not have ferroelectrically active cations. The distribution of the oxygen vacancy induced electrons are quite different in the three materials even though they have similar structures. In oxygen deficient LiNbO_3−*δ*_ (*δ* = 0.083/f.u.), electrons are itinerant, while in ZnTiO_3−*δ*_ and ZnSnO_3−*δ*_ (*δ* = 0.083/f.u.) the electrons are localized. These results provide guidance for the application of oxygen vacancies in LiNbO_3_-type ferroelectric material devices.

## Abstract 2

LiNbO_3_-type ferroelectric oxides, as an important class of non-centrosymmetric compounds, have received great attention due to their important and rich properties.

## Graphical abstract

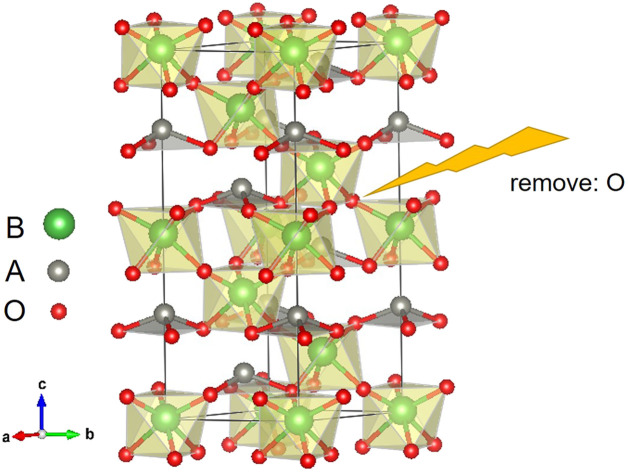

## 1. Introduction

Oxygen vacancies are one of the most common point defects in oxides.^1–5^ For example, oxygen vacancies are one of the basic and inherent defects of perovskite oxides and are widely present in perovskite oxides. The presence of oxygen vacancies may significantly change their physical and chemical properties, which can be used to achieve new functionalities.^6–9^ Experimental and theoretical studies of oxygen vacancies in prototypical perovskite oxides have been reported, such as SrTiO_3_, BaTiO_3_, PbTiO_3_*et al.*^10–12^ Usually, it is an effective way to dope electrons in oxides by oxygen vacancies. Oxygen vacancies can be introduced into perovskite oxides through various processes such as growth, annealing, and redox reactions.^13^ One oxygen vacancy contribute two electrons, which distribute in the materials depend on the properties. For example, oxygen vacancy doping in SrTiO_3_ can lead to the transition from insulator to metal.^14^ However, electrons may be trapped.^15^ Isolated oxygen vacancies may form defect states which localize conduction electrons.^16–18^

Especially, in polar oxides, the existence of oxygen vacancies has crucial effects on the polarization.^19,20^ The studies of oxygen vacancies in perovskite ferroelectrics such as BaTiO_3_ and PbTiO_3_ have been widely reported.^21,22^ Cheng *et al.* reported the transformation of oxygen vacancies from an isolated state to a clustered state in LiNbO_3_ single crystal, making controlling the oxygen vacancy state a promising option.^23^ However, apart from LiNbO_3_, there are not many studies on oxygen vacancies in other important polar oxides with LiNbO_3_ type (LN type) structure. Recently, more and more LN-type structural materials have been synthesized, such as ZnSnO_3_,^24,25^ ZnTiO_3_,^26^ ZnPbO_3_,^27^ PbNiO_3_.^28^ Therefore, it is desirable to study the properties of oxygen vacancies and their effects on polar displacements.

For this work, we chose three representative LN-type ABO_3_ ferroelectrics LiNbO_3_, ZnTiO_3_ and ZnSnO_3_ to study the influence of oxygen vacancy on polarization. ZnSnO_3_ does not have ferroelectrically active cations, while ZnTiO_3_ has the ferroelectrically active cation Ti^4+^. Compared with ZnTiO_3_ and ZnSnO_3_, LiNbO_3_ has different chemical valence on both A and B sites. Therefore, the comparison of these three representative LN-type ferroelectrics is helpful to understand the impact of the oxygen vacancy on other LN-type ferroelectrics. Our study shows that, in the three compounds, polar displacements persists at a certain level of oxygen vacancy concentration, but exhibit completely different behaviors. In LiNbO_3_, the electrons induced by oxygen vacancy are itinerant. However, in ZnTiO_3_ and ZnSnO_3_ they are localized.

## 2. Methods and computational details

All first-principles calculations were performed with the Quantum ESPRESSO code^29^ within the local density approximation (LDA). We also used the Perdew–Burke–Ernzerhof functional revised for solids (GGA-PBEsol)^30^ to verify our main calculation results. The key results of our calculations did not change qualitatively due to different exchange correlation functions. Detailed results are given in Section IV of the ESI.†^31^ The cutoff energy was set to 650 eV. The atomic positions in all structures were relaxed until forces were converged to less than 10 meV Å^−1^. The convergence value of the self-consistent calculation was 10^−5^ eV.

**Fig. 1:**
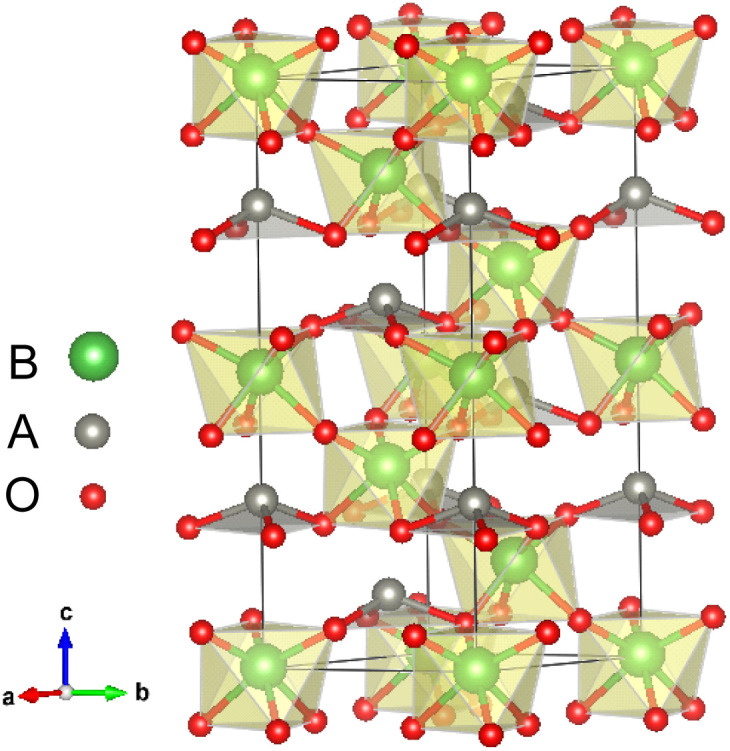
Atomic structures of LN-type ABO_3_ oxides: polarized *R*3*c* structure.

The LN type structure is closely related to the perovskite oxide type structure and both have three-dimensional corner-sharing BO_6_ octahedrons. The octahedral rotation of the LN-type ferroelectric with the *R*3*c* structure is *a*^−^*a*^−^*a*^−^ in Glazer notation. The [111] direction in the pseudo cubic lattice of the Pv-type structure corresponds to the hexagonal *c*-direction of the LN-type structure. In LN-type compounds, along the *c* direction of the hexagonal structure, there is a relative displacement of cations relative to the anion layer (oxygen layer), leading to the occurrence of spontaneous polarization, as shown in Fig. 1. We choose the hexagonal structure (30 atoms) of LN-type ferroelectric materials to perform pristine bulk calculations. In Table 1, we list the calculated and experimental lattice parameters of the hexagonal unit cells of these three materials. We can see that our calculated results are consistent with the experimental results.

**Table 1:** Hexagonal structural parameters for LN-type LiNbO_3_, ZnTiO_3_ and ZnSnO_3_

Material		*a*/Å	*c*/Å
LiNbO_3_	Expt.^33^	5.147	13.856
(*R*3*c*)	Present	5.067	13.679
ZnTiO_3_	Expt^26^	5.09452	13.7177
(*R*3*c*)	Present	5.019	13.558
ZnSnO_3_	Expt^24^	5.2622	14.0026
(*R*3*c*)	Present	5.246	13.900

We use supercell calculations to simulate charge neutral oxygen vacancies. To simulate oxygen-deficient LiNbO_3−*δ*_, ZnTiO_3−*δ*_, and ZnSnO_3−*δ*_, we start from the *R*3*c* structure of pristine LN-type ferroelectric and remove one charge-neutral oxygen atom. In the *R*3*c* LN-type ferroelectric structure, all oxygen atoms positions are equivalent and there is only one Wyckoff position. To study the distribution of electrons induced by the oxygen vacancy, we perform calculations on a supercell of 59-atom, which corresponds to an oxygen vacancy concentration of 0.083/f.u. and electron doping concentration of 0.17 e/f.u. 4 × 2 × 2 and 12 × 6 × 6 Monkhorst–Pack *k*-point grids are used for the calculation of structural relaxation and density of states (DOS), respectively. We perform spin polarization calculations in pristine LiNbO_3_, ZnTiO_3_ and ZnSnO_3_, as well as oxygen-deficient LiNbO_3−*δ*_, ZnTiO_3−*δ*_ and ZnSnO_3−*δ*_. As shown in Fig. S1 in the ESI,†^31^ DOS did not show any magnetization in our calculations. Therefore, we sum the two spins when calculating DOS. We fully relax the structure including lattice constants and internal coordinates to obtain the ground state structure. Using the VESTA software package,^32^ we visualize the crystal structure and iso-surfaces of the charge distribution.

With oxygen vacancy, the polarization cannot be calculated using the Berry phase method due to the existence of free charge. Therefore, we focus on analyzing the polar displacement of cations and anions. For the polarized *R*3*c* structure, taking LiNbO_3_ as an example as shown by Fig. 1, the Li atom at A site is surrounded by three O atoms in the same plane. The relative displacement between the Li atom and the center of the three oxygen atoms in the *c*-axis direction is recorded as *δ*_Li−O_. While the relative displacement between the Nb atom and the center of the surrounding six O atoms along the *c*-axis direction is recorded as *δ*_Nb−O_. When an oxygen atom is removed, there is one Li atom with only two nearest O atoms, and two Nb atoms surrounded by the five nearest O atoms.

## 3. Results and discussion

**Fig. 2:**
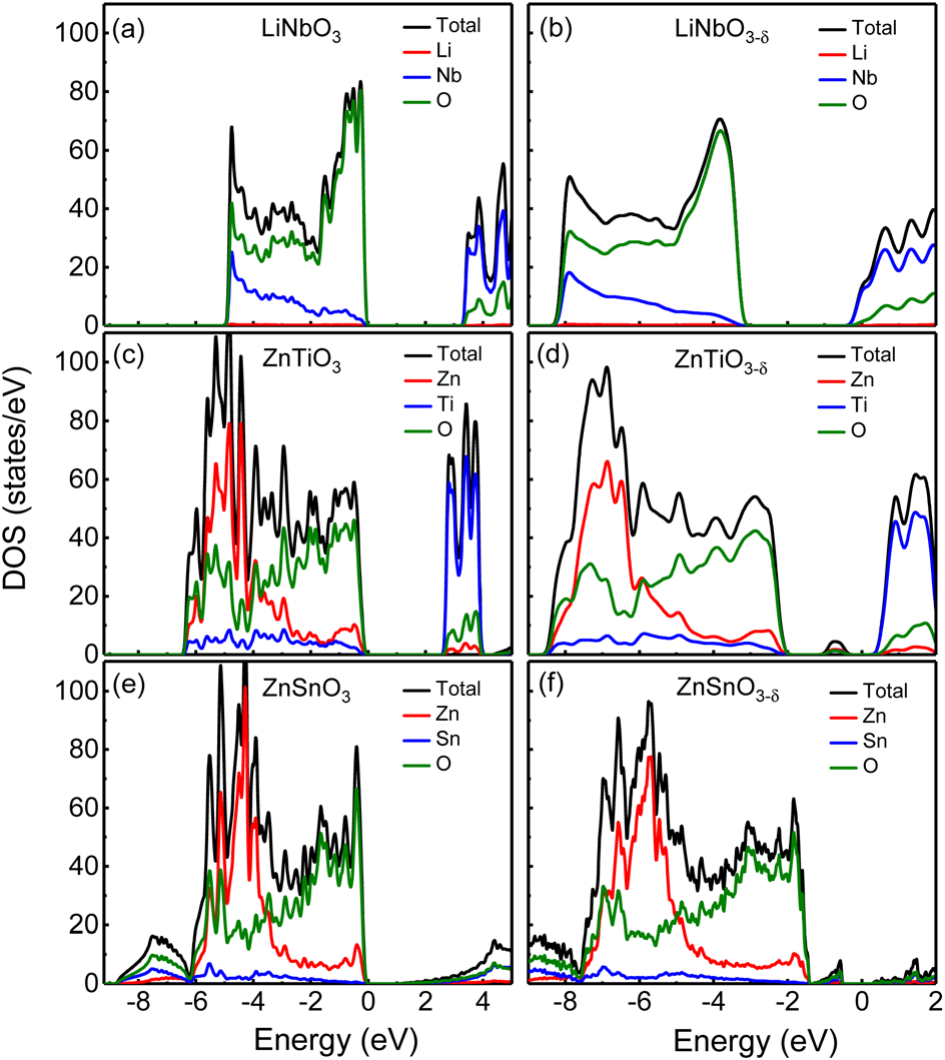
The density of states of (a) LiNbO_3_, (c) ZnTiO_3_ and (e) ZnSnO_3_ without oxygen vacancies, respectively; the density of states of oxygen-deficient (b) LiNbO_3−*δ*_, (d) ZnTiO_3−*δ*_, (f) ZnSnO_3−*δ*_ (*δ* = 0.083/f.u.), respectively. The Fermi level is set 0.

First, we analyze the electronic structures of the three materials without oxygen vacancies. Fig. 2 shows the total density of states without oxygen vacancies of the *R*3*c* structure of (a) LiNbO_3_, (c) ZnTiO_3_, and (e) ZnSnO_3_. The partial density of states (PDOS) of these three materials are shown in Fig. S2 in the ESI.†^31^ For LiNbO_3_, as shown by the black curve of the DOS in Fig. 2(a), we can see two peaks near the top and bottom of the valence band (VB). Fig. S2(a) in the ESI†^31^ shows that the peak near the top of VB is mainly contributed by O-p orbitals, while the peak near the bottom of VB is contributed by Nb-d orbitals and O-p orbitals. The origin of the Nb-d orbital and O-*p* peaks is the less dispersive band at the bottom of the VB (about −5 eV) of LiNbO_3_.^34^ The bottom of conduction band (CB) in LiNbO_3_ is mainly composed of the d orbitals of Nb atoms.

It can be seen from the density of states of the green curves in Fig. 2(c) and (e) that the tops of the VB (from −5 eV to the Fermi level) of ZnTiO_3_ and ZnSnO_3_ are mainly occupied by oxygen orbitals and Zn orbitals as shown by the green and red curves respectively. The PDOS in Fig. S2 in the ESI†^31^ shows that the peak near the top of VB is contributed by the O-p orbital. For both ZnTiO_3_ and ZnSnO_3_, a relatively sharp DOS peak can be seen near −5 eV, which is a typical local property of the A-site Zn band.^35^ However, in LiNbO_3_, the A-site Li doesn't contribute to the VB as shown by the red curves in Fig. 2(a). Fig. S2(b) and (c) in the ESI†^31^ shows that the peak is contributed by Zn-d orbitals. The Zn-d orbitals are weakly hybridized with the O p band, which can be understood from the fact that Zn^2+^ (d^10^) is a fully occupied 3d orbital. It is also demonstrated by the calculated Born effective charge (BEC) of Zn in ZnTiO_3_ and ZnSnO_3_ materials close to its nominal charge +2, as shown in Table 2. It can be seen from the density of states of the blue curve in Fig. 2(c) and the PDOS in Fig. S2(b) in the ESI†^31^ that the bottom of CB in ZnTiO_3_ is mainly contributed by Ti-d orbitals. From Fig. 2(e) and Fig. S2(c) in the ESI,†^31^ it can be seen that the bottom of the CB of ZnSnO_3_ is mainly composed of the s orbitals of Sn atoms and p orbitals of O atoms.

**Table 2:** Born effective charges Z* of LiNbO_3_, ZnTiO_3_ and ZnSnO_3_

Material		Z* (*e*)		
		*Z*_*xx*_	*Z*_*yy*_	*Z*_*zz*_
LiNbO_3_	Li	1.17	1.17	1.01
(*R*3*c*)	Nb	7.35	7.35	7.02
ZnTiO_3_	Zn	2.49	2.49	2.20
(*R*3*c*)	Ti	6.09	6.09	5.34
ZnSnO_3_	Zn	2.32	2.32	2.13
(*R*3*c*)	Sn	4.18	4.18	4.21

By comparing LiNbO_3_ and ZnTiO_3_, both B-sites are ferroelectric active cations with partially occupied d orbitals, while A-site cations are very different. In LiNbO_3_, the contribution of Li atoms to VB is negligible. In ZnTiO_3_, Zn atoms contribute greatly to VB. Another obvious difference is the hybridization of B-site cations and oxygen anions. In LiNbO_3_, Nb atoms have more contribution to VB compared with Ti.

Compared with ZnTiO_3_, the contribution of Sn atoms to VB in ZnSnO_3_ is smaller, as shown by the blue curves in Fig. 2(c) and (e). The BEC can reflect the covalency of each atom's bonding environment relative to its nominal ionic value.^36^ As shown in Table 2, the BEC of Nb in LiNbO_3_ deviates most from the nominal charge (+5). The BEC of Ti in ZnTiO_3_ deviates less from the nominal charge (+4). The BEC of Sn in ZnSnO_3_ is almost consistent with the nominal charge (+4), and the deviation is very small. Furthermore, it is also evident from Table 2 that cations with d^0^ (Nb^5+^ and Ti^4+^) electron configuration have larger BEC values than the corresponding nominal charges than cations with d^10^ (Zn^2+^ and Sn^4+^) electron configuration.

We next compare the electronic structures of oxygen-deficient LiNbO_3−*δ*_, ZnTiO_3−*δ*_ and ZnSnO_3−*δ*_ (*δ* = 0.083/f.u.), as shown in Fig. 2(b), (d) and (f). In Fig. S5 in the ESI,†^31^ we provide a detailed comparison of the band gaps calculated by LDA, HSE06, and PBEsol for bulk LiNbO_3_, ZnTiO_3_, and ZnSnO_3_. In Fig. 2(a), the band gap of LiNbO_3_ we calculated is 3.3 eV, which is close to the experimental value of 3.78 eV of LiNbO_3_.^37^ This is consistent with previous calculations and the gap results are reliable.^38^ However, we find that for oxygen-deficient LiNbO_3−*δ*_ (*δ* = 0.083/f.u.), the Fermi level moves into the CB, and no additional localized states appear in the band gap, as shown in Fig. 2(b). The oxygen-deficient LiNbO_3−*δ*_ is a conductor with electrons distribute overall the system. While ZnTiO_3−*δ*_ and ZnSnO_3−*δ*_ are insulators with electrons localized around the oxygen vacancy. For oxygen-deficient ZnTiO_3−*δ*_ and ZnSnO_3−*δ*_ (*δ* = 0.083/f.u.), we can see localized states in the band gap at 0.50 and 0.75 eV below the conduction band minimum (CBM), as shown by Fig. 2(b) and (f). The distribution of defect states can be clearly seen in Fig. S3 in the ESI.†^31^

**Fig. 3:**
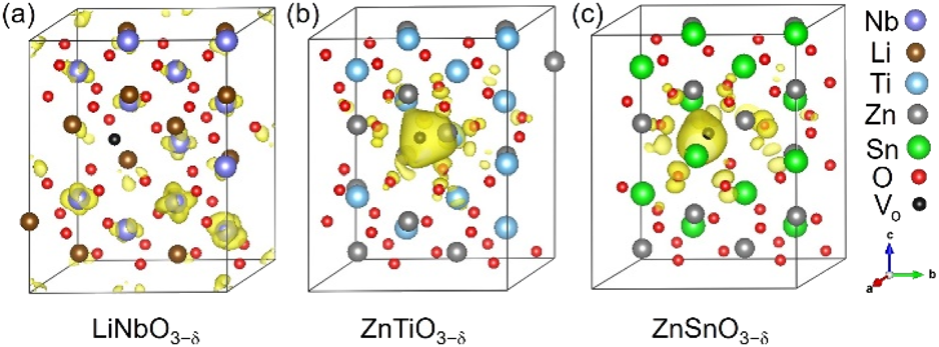
Spatial distributions of oxygen vacancy doping electrons in oxygen-deficient (a) LiNbO_3−*δ*_, (b) ZnTiO_3−*δ*_ and (c) ZnSnO_3−*δ*_ (*δ* = 0.083/f.u.), respectively. The yellow area represents the distribution of electrons. The iso-surfaces correspond to a charge density of 0.004 e bohr^−3^.

Each oxygen vacancy contributes two electrons to the system, and then we study the distribution of the electrons. In Fig. 2(b), our integrated value for the total DOS of oxygen-deficient LiNbO_3−*δ*_ (*δ* = 0.083/f.u.) from the band gap to the Fermi level is 2, and the two electrons provided by the oxygen vacancy occupy the conduction band. In Fig. 2(d) and (f), for oxygen-deficient ZnTiO_3−*δ*_ and ZnSnO_3−*δ*_ (*δ* = 0.083/f.u.), we integrate the total DOS of the localized states in the band gap to get a value of 2, which means that two electrons occupy the defect state. The spatial distribution of oxygen vacancy doping electrons in these three materials can be seen more clearly from Fig. 3. For oxygen-deficient LiNbO_3−*δ*_ (*δ* = 0.083/f.u.), since the conduction band is contributed by the Nb-d orbital, electrons are mainly distributed uniformly on Nb sites, which is clearly reflected by Fig. 3(a). The localization of the electrons in ZnTiO_3−*δ*_ and ZnSnO_3−*δ*_ (*δ* = 0.083/f.u.) is illustrated in Fig. 3(b) and (c). In oxygen-deficient ZnTiO_3−*δ*_ (*δ* = 0.083/f.u.), electrons around oxygen vacancies are mainly distributed on the nearest neighbor Zn atom. In oxygen-deficient ZnSnO_3−*δ*_ (*δ* = 0.083/f.u.), electrons around oxygen vacancies are mainly distributed on the nearest neighbor Sn atom. We can see that the electrons localize near the oxygen vacancies and have the greatest impact on nearby atoms.

**Fig. 4:**
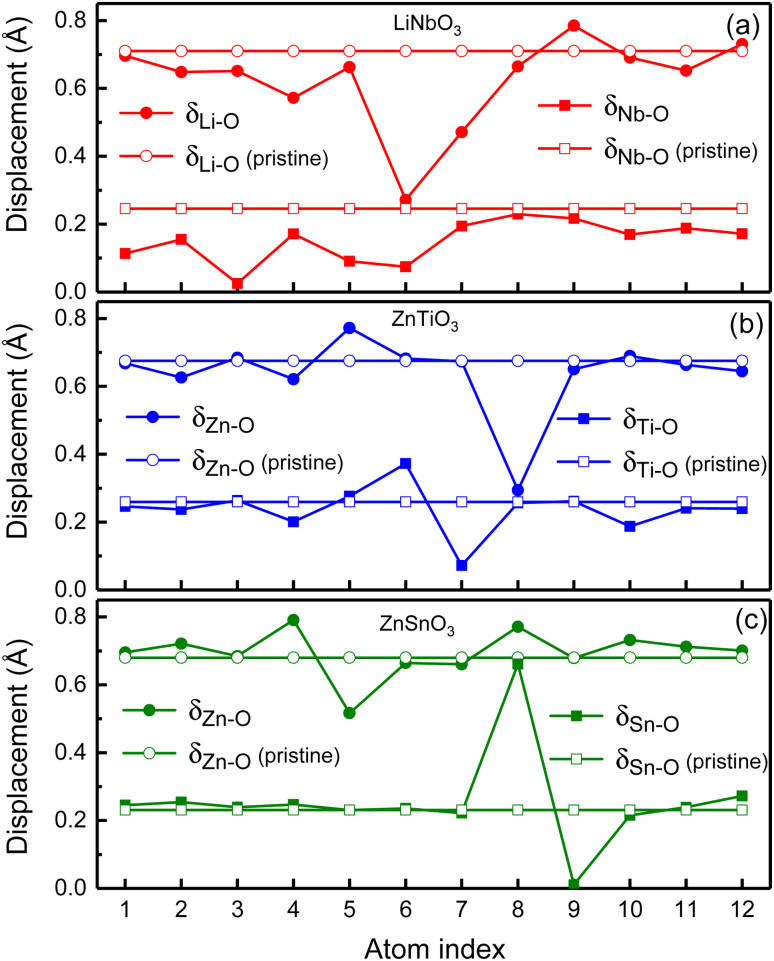
Polar displacements of each Li atom, Nb atom, Zn atom, Ti atom and Sn atom in oxygen-deficient (a) LiNbO_3−*δ*_, (b) ZnTiO_3−*δ*_ and (c) ZnSnO_3−*δ*_ (*δ* = 0.083/f.u.), respectively. Solid circles and squares are for the oxygen-deficient structures while the open circles and squares are for the pristine structures.

In order to illustrate the influence of oxygen vacancies on polarization, we study the relative displacements between cations and anions of oxygen-deficient LiNbO_3−*δ*_, ZnTiO_3−*δ*_ and ZnSnO_3−*δ*_ (*δ* = 0.083/f.u.) which are shown by the solid circles and squares in Fig. 4. To show the difference of relative displacement with and without oxygen vacancies, we compare them with the pristine structures, which are represented by the open circles and squares in Fig. 4.

For LiNbO_3−*δ*_ (*δ* = 0.083/f.u.), the displacements of Li atoms and Nb atoms are drastically reduced, and the change in the displacement of Nb atoms is more obvious as shown in Fig. 4(a). We could see that the average displacement of *δ*_Nb−O_ in oxygen-deficient LiNbO_3−*δ*_ (*δ* = 0.083/f.u.) is 0.15 Å, which is much smaller than the displacement of the pristine LiNbO_3_ (*δ*_Nb−O_ is 0.24 Å), and the average displacement is reduced by about 40%. The average displacement of *δ*_Li−O_ also decreases. Our results are consistent with previous studies.^39^ The reduction of the polar displacement is because of the electrons induced by the oxygen vacancies. The uniformly distributed free electrons in the whole system on the Nb-d orbitals has screening effects on the long-range Coulomb interaction which is responsible to the polarization.

For oxygen-deficient ZnTiO_3−*δ*_ and ZnSnO_3−*δ*_ (*δ* = 0.083/f.u.), due to the localization of the oxygen vacancy induced electrons, we cannot see overall reduction of the polar displacement as that in LiNbO_3−*δ*_ (*δ* = 0.083/f.u.). There is only obvious changing of the polar displacements on atoms that are close to the oxygen vacancy. For oxygen-deficient ZnTiO_3−*δ*_ (*δ* = 0.083/f.u.) as shown in Fig. 4(b), the nearest neighbor Zn atom of the oxygen vacancy is labeled 8, and the second nearest neighbor Zn atom is labeled 5. The nearest neighbor Ti atom of the oxygen vacancy is labeled 6, and the second nearest neighbor Ti atom is labeled 7. As shown in Fig. 4(c), for oxygen-deficient ZnSnO_3−*δ*_ (*δ* = 0.083/f.u.), the nearest neighbor Zn atom is labeled 8, and the second nearest neighbor Zn atom is labeled 5. The nearest neighbor Sn atom is labeled 8, and the second nearest neighbor Sn atom is labeled 9. Except for the large changes in the nearest neighbor and second nearest neighbor atomic displacements of the oxygen vacancy, the atomic displacements at other positions are close to the displacements of the pristine ZnTiO_3_ and ZnSnO_3_. This is because the localization of the electrons does not have screening effect in the whole structure.

Finally, we calculate the formation energies of charge neutral oxygen vacancy of LiNbO_3−*δ*_, ZnTiO_3−*δ*_ and ZnSnO_3−*δ*_. We remove a single oxygen atom in the supercell. The oxygen vacancy formation energy is defined as,^40^

1 Δ*E*_form_ = *E*_defect_(*V*_O_) − *E*_ideal_ + *μ*_O_,

where *E*_defect_(*V*_O_) and *E*_ideal_ are the total energies of the supercell with one oxygen vacancy and the pristine supercell without oxygen vacancy respectively. *μ*_O_ is the chemical potential of oxygen, which depends on the thermodynamic conditions of the system which is half the total energy of the oxygen molecule.^41^

**Fig. 5:**
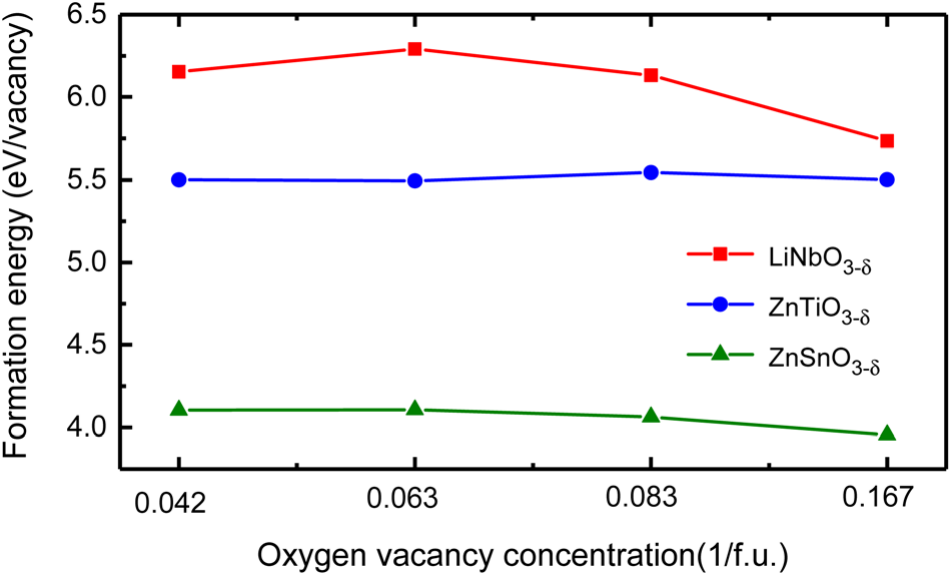
The oxygen vacancy formation energy of LiNbO_3−*δ*_, ZnTiO_3−*δ*_ and ZnSnO_3−*δ*_ (*δ* = 0.042/f.u., 0.063/f.u., 0.083/f.u. and 0.167/f.u.) changes as a function of oxygen vacancy concentration.

We use different supercells to calculate the oxygen vacancy formation energy at different concentrations. We consider 30 atoms, 60 atoms, and 120 atoms hexagonal supercells, as well as 80 atoms rhombohedral supercell, and schematics of these structures are in Fig. S4 in ESI.†^31^ We remove one charge-neutral oxygen atom in these structures and the oxygen vacancy concentrations are *δ* = 0.167/f.u., 0.083/f.u., 0.042/f.u. and 0.063/f.u., respectively. Fig. 5 shows the oxygen vacancy formation energy as a function of oxygen vacancy concentration.

We see that the change of oxygen vacancy concentration has no significant impact on the formation energy of oxygen vacancies. The formation energy of LiNbO_3_ is relatively close to the neutral oxygen vacancy formation energy of tetragonal BaTiO_3_ of 6.35 eV.^42^ The formation energy of LiNbO_3_ and ZnTiO_3_ are much higher than that of ZnSnO_3_. Probably it is due to the covalent bonding between Nb–O and Ti–O are stronger than Sn–O as shown in Fig. 2. Even though the formation energy of oxygen vacancies in LiNbO_3−*δ*_ is higher, oxygen vacancies were observed in the experiments.^43,44^ There have been experimental reports on the study of oxygen vacancies in ZnSnO_3_.^45^ It is reported that the oxygen vacancies in ferroelectric ZnSnO_3_ nanowires can serve as exciton capture centers, and the deep energy levels serve as donor bands, enhancing electron lifetime and effectively promoting electrons to reach CB under light irradiation. We can expect that similar studies in LN-type ferroelectric oxides can expand our understanding of the behavior of oxygen vacancies and provide guidance for the application of specific oxygen vacancy properties in new devices.

## 4. Conclusion

In conclusion, we use first-principles calculations to study the effect of oxygen vacancy doping on three representative LN-type ferroelectric oxide materials. LiNbO_3_ and ZnTiO_3_ have ferroelectrically active cations Nb^5+^ and Ti^4+^, while ZnSnO_3_ does not have ferroelectrically active cations. With comparison, we study the distribution of the oxygen vacancy induced electrons. Our results show that in oxygen-deficient LiNbO_3−*δ*_, electrons are itinerant. Although the polar displacement is reduced by oxygen vacancy doping, polar displacements and conductivity can coexist in LiNbO_3−*δ*_. However, in oxygen-deficient ZnTiO_3−*δ*_ and ZnSnO_3−*δ*_ electrons are localized around the oxygen vacancy. To realize conducting ferroelectric in ZnTiO_3_ and ZnSnO_3_, doping oxygen vacancy probably is not an effective way.

## Conflicts of interest

There are no conflicts to declare.

## Supplementary Material

RA-014-D4RA00833B-s001
