# Supplementary material for: First-principles study of oxygen vacancies in LiNbO3-type ferroelectrics
Source: RSC Adv. 2024 Mar 18;14(13):9169–74. doi: 10.1039/d4ra00833b (PMC10946246; doi:10.1039/d4ra00833b)
Supplement: RA-014-D4RA00833B-s001 [file RA-014-D4RA00833B-s001.pdf]

Electronic Supplementary Material (ESI) for RSC Advances.
This journal is © The Royal Society of Chemistry 2024

SUPPLEMENTAL MATERIAL

First-principles study of oxygen vacancy in $LiNbO_3$-type ferroelectrics

Jing Li, Xiaohui Liu*

School of Physics, Shandong University, Ji'nan 250100, China

I. Spin-polarized calculations

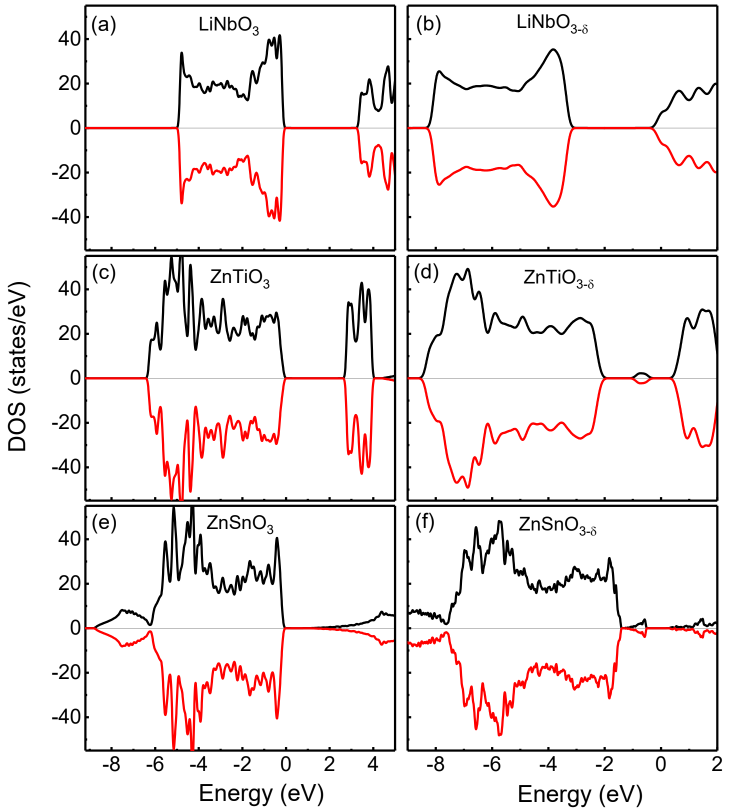

Fig. S1 Density of states of (a) $LiNbO_3$, (c) $ZnTiO_3$ and (e) $ZnSnO_3$ without oxygen vacancies, respectively; density of states of oxygen-deficient (b) $LiNbO_{3-\delta}$, (d) $ZnTiO_{3-\delta}$, (f) $ZnSnO_{3-\delta}$ (δ=0.083/f.u.), respectively. The black and red lines are the spin-up and spin-down densities of states, respectively. The Fermi level is set 0.

We present spin polarization calculations for pristine $LiNbO_3$, $ZnTiO_3$ and $ZnSnO_3$, as well as oxygen-deficient $LiNbO_{3-\delta}$, $ZnTiO_{3-\delta}$ and $ZnSnO_{3-\delta}$, as shown in Fig. S1. In our calculations, DOS does not show any magnetization.

II. Density of states

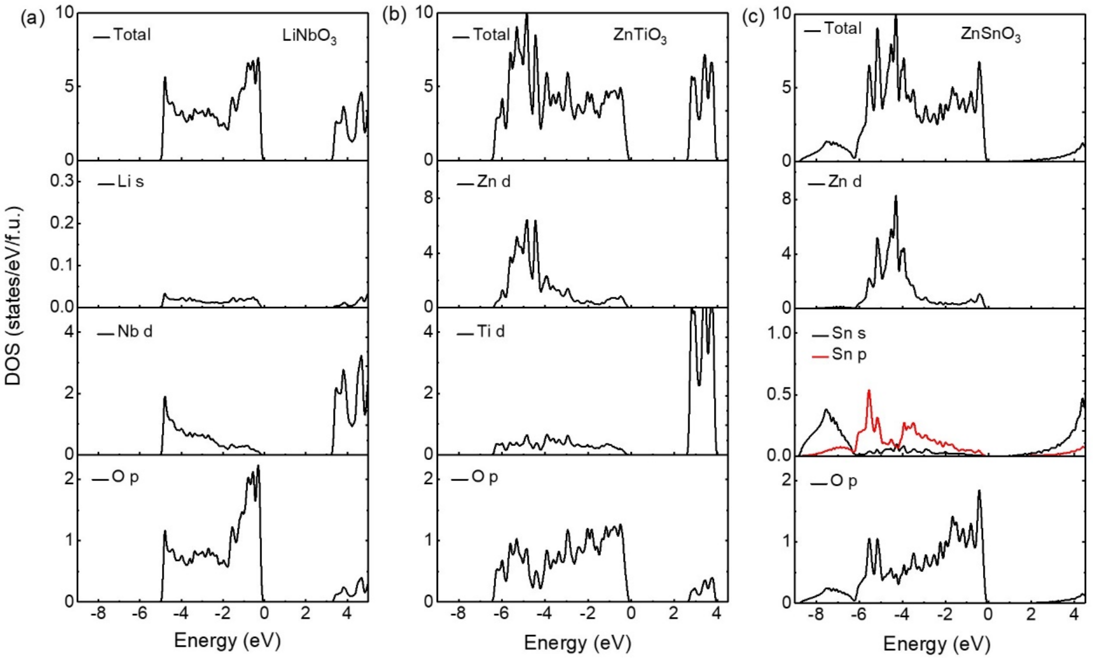

Fig. S2 Total and partial density of states of (a) $LiNbO_3$, (b) $ZnTiO_3$ and (c) $ZnSnO_3$. The Fermi level is set at 0 eV.

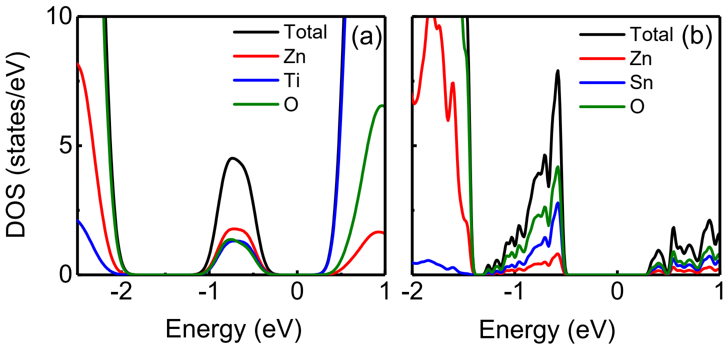

Fig. S3 (a) and (b) are the density of states of defect states near the Fermi level in oxygen-deficient $ZnTiO_{3-\delta}$ and $ZnSnO_{3-\delta}$ (δ=0.083/f.u.), respectively.

III. Supercell structures

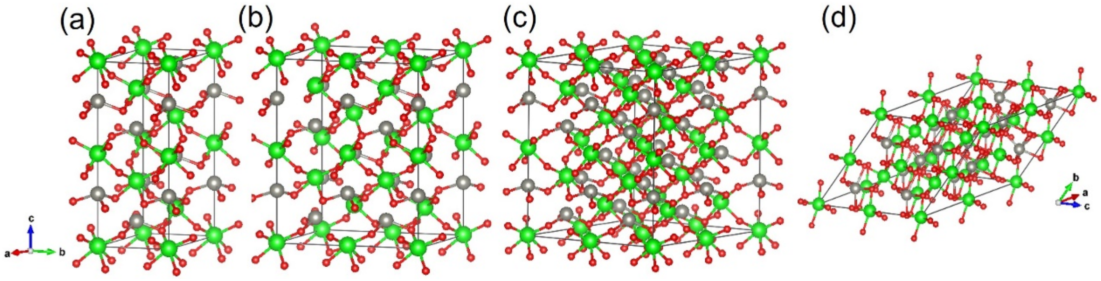

Fig. S4 Atomic structures of LN-type ABO_3 oxides, Gray, A; Green, B; Red, O. (a), (b) and (c) are hexagonal supercell structures of 30 atoms, 60 atoms and 120 atoms respectively; (d) is a rhombohedral supercell structure of 80 atoms.

IV. PBEsol calculations

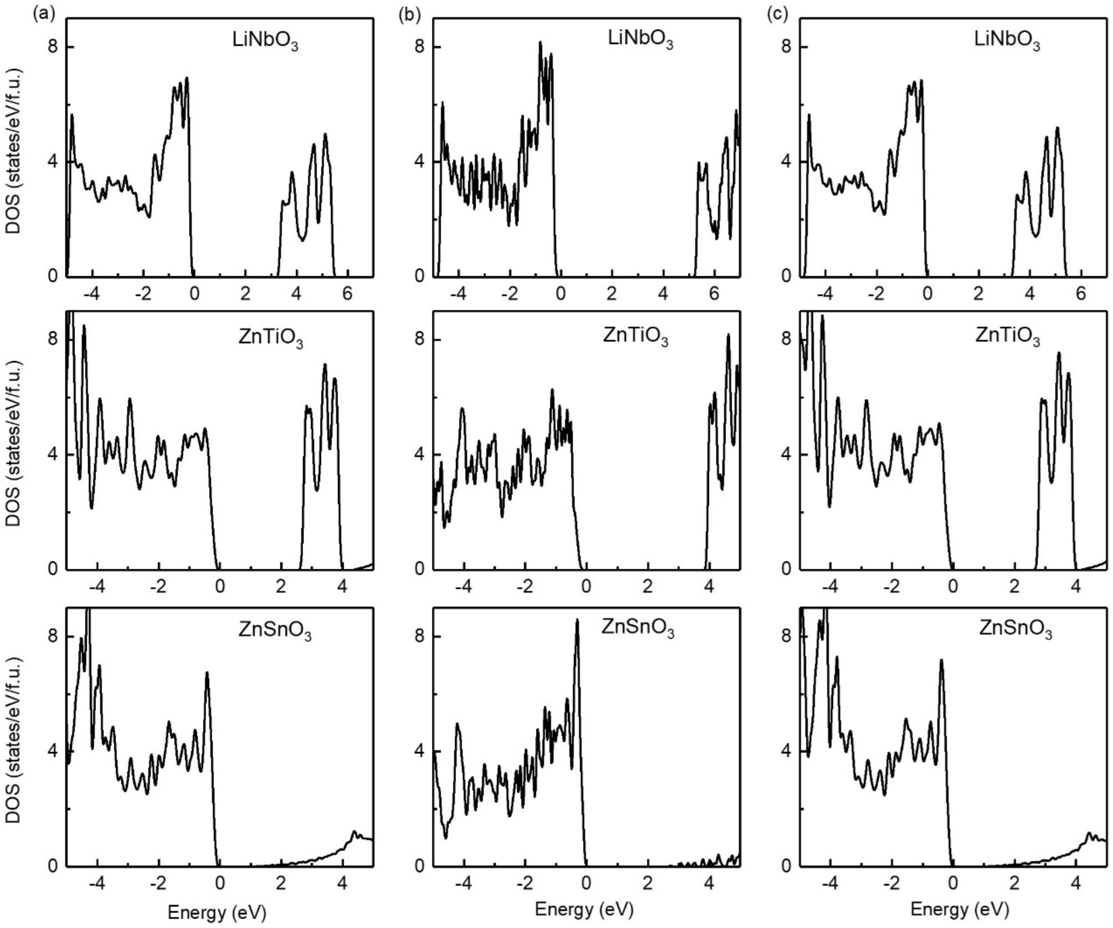

Fig. S5 Total state density of bulk $LiNbO_3$, $ZnTiO_3$ and $ZnSnO_3$. (a), (b) and (c) were calculated using LDA, HSE06 and PBEsol, respectively. The Fermi level is set at 0 eV.

In Fig. S5, we calculate the total density of states of bulk $LiNbO_3$, $ZnTiO_3$, and $ZnSnO_3$ bulk using LDA, HSE06[1] and PBEsol. For $LiNbO_3$, the experimental band gap of $LiNbO_3$ is 3.78 eV, the previously reported band gaps calculated by HSE06 are 5.4 eV, 5.9 eV and 6.2 eV.[2] We use HSE06 to calculate the band gap of $LiNbO_3$ to be 5.2 eV. The band gaps calculated by LDA and PBEsol range from 3.2 eV to 3.3 eV. The band gaps calculated by LDA and PBEsol are closer to the experimental results. For $ZnTiO_3$, the experimental band gap of ZnTiO3 is 3.18 eV.[3] HSE06 calculates the band gap of $ZnTiO_3$ to be 3.9 eV, the band gap calculated by LDA and PBEsol is around 2.6 eV. Similarly, the band gaps calculated by LDA and PBEsol are closer to the experimental values. For $ZnSnO_3$, the experimental band gap of $ZnSnO_3$ is 2.6–3.4 eV.[4] The $ZnSnO_3$ band gap calculated by HSE06 is 2.2 eV, which is the closest to the experimental value among the three. The band gaps calculated by LDA and PBEsol are at 1.0 eV to 1.1eV. Hybrid functionals such as HSE06 can overestimate the lattice constants and atomic distortions associated with ferroelectricity.[5],[6] On the other hand, the application of hybrid functionals is still quite expensive since calculations of point defects often require large supercells. The PBEsol functional shows high accuracy in predicting structures, such as the $BaTiO_3$ rhombohedral phase.[7] To this end, we use PBEsol to calculate the supercell of oxygen vacancy defects, examining key results of our calculations.

We use PBEsol to check the key results of our calculations. In Table S1, we list the lattice parameters of these three materials calculated with LDA and PBEsol. By comparing with experimental values, we can see that the PBEsol calculation results are in better agreement with the experimental results.

TABLE SI. Hexagonal structural parameters for LN-type $LiNbO_3$, $ZnTiO_3$ and $ZnSnO_3$.

Material		a / Å	c / Å
$LiNbO_3$	Expt.[8]	5.147	13.856
($R3c$)	LDA	5.067	13.679
($R3c$)	PBEsol	5.134	13.828
$ZnTiO_3$	Expt.[9]	5.09452	13.7177
($R3c$)	LDA	5.019	13.558
($R3c$)	PBEsol	5.070	13.701
$ZnSnO_3$	Expt.[10]	5.2622	14.0026
($R3c$)	LDA	5.246	13.900
($R3c$)	PBEsol	5.284	14.011

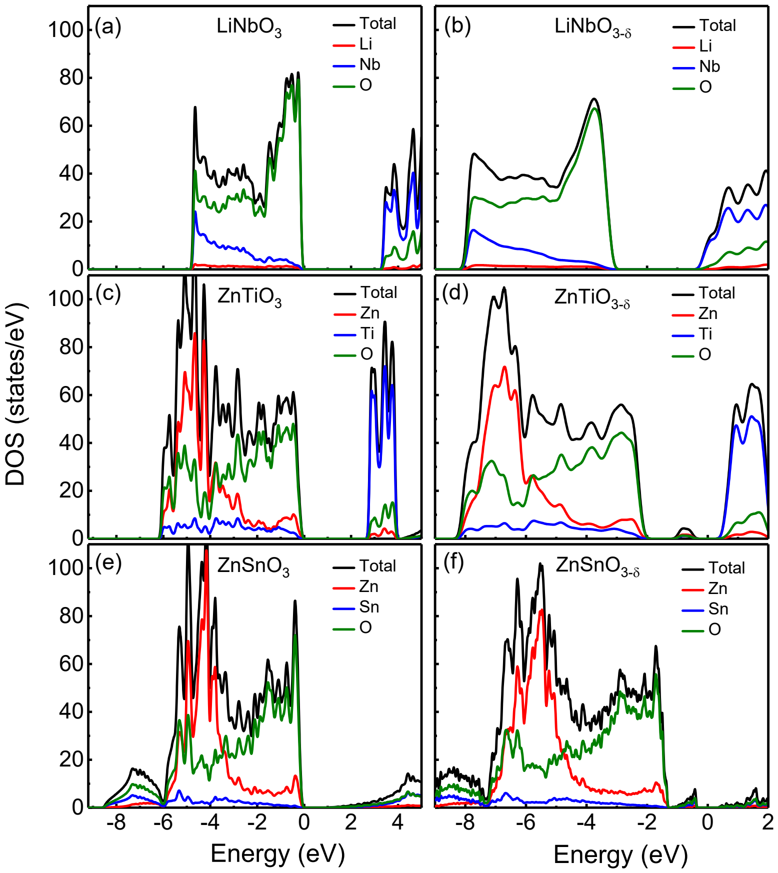

Fig. S6 Density of states of (a) $LiNbO_3$, (c) $ZnTiO_3$ and (e) $ZnSnO_3$ without oxygen vacancies, respectively; The density of states of oxygen-deficient (b) $LiNbO_{3-\delta}$, (d) $ZnTiO_{3-\delta}$, (f) $ZnSnO_{3-\delta}$ (δ=0.083/f.u.), respectively. The Fermi level is set 0.

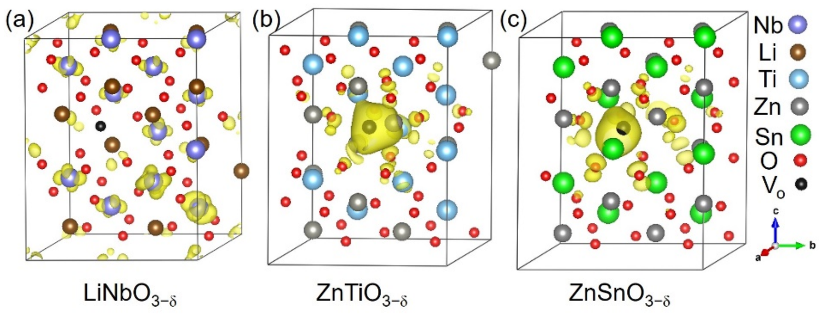

Fig. S7 Spatial distributions of oxygen vacancy doping electrons in oxygen-deficient (a) $LiNbO_{3-\delta}$, (b) $ZnTiO_{3-\delta}$ and (c) $ZnSnO_{3-\delta}$ (δ=0.083/f.u.), respectively. The yellow area represents the distribution of electrons. The iso-surfaces correspond to a charge density of 0.004 e/bohr^3.

In Fig. S6, we show the density of states calculated using PBEsol. (a), (b) and (c) are the total state densities of $LiNbO_3$, $ZnTiO_3$ and $ZnSnO_3$ without oxygen vacancies, respectively. (b), (d) and (f) are the density of states of oxygen-deficient $LiNbO_{3-\delta}$, $ZnTiO_{3-\delta}$ and $ZnSnO_{3-\delta}$ (δ=0.083/f.u.) respectively.

Fig. S7 shows the spatial distribution results of oxygen vacancy doping electrons in oxygen defects calculated using PBEsol.

We use PBEsol to check our key results. No significant changes were found. In oxygen deficient $LiNbO_{3-\delta}$ (δ=0.083/f.u.), electrons are itinerant, while in $ZnTiO_{3-\delta}$ and $ZnSnO_{3-\delta}$ (δ=0.083/f.u.) the electrons are localized.

[1] J. Heyd, G. Scuseria, and M. Ernzerhof, *J. Chem. Phys.*, 2003, **118**, 8207
[2] A. Riefer, M. Friedrich, S. Sanna, U. Gerstmann, Arno Schindlmayr, and W. G. Schmidt, *Phys. Rev. B*, 2016, **93**, 075205
[3] J. Yu, N. Li, L. Zhu, X. Xu, *J. Alloys Compd.*, 2016, **681**, 88–95.
[4] F.-Y. Wu, J.-W. Li, Y. Qi, W.-T. Ding, Y.-Y. Guo, Y.-W. Zhou, Acta Metall. Sin. (Engl. Lett.) 2016, 29, 827.
[5] R. A. Evarestov and A. V. Bandura, *J. Comput. Chem.*, 2012, **33**, 1123
[6] N. Tsunoda, Y. Kumagai, and F. Oba, *Phys. Rev. Mater.*, 2019, **3**, 114602
[7] G. Gebreyesus, Lorenzo Bastonero, Michele Kotiuga, Nicola Marzari, and Iurii Timrov, *Phys. Rev. B*, 2023, **108**, 235171
[8] S. C. Abrahams, P. Marsh, *Acta Crystallogr.*, Sect. B: Struct. Sci., 1986, **42**,61-68
[9] Y. Inaguma, A. Aimi, Y. Shirako, D. Sakurai, D. Mori, H. Kojitani, M. Akaogi and M. Nakayama, *J. Am. Chem. Soc.*, 2014, **136** (7), 2748-2756
[10] Y. Inaguma, M. Yoshida and T. Katsumata, *J. Am. Chem. Soc.*, 2008, **130**, 6704-6705
